# Structural Safety of the Steel Hall under Dynamic Excitation Using the Relative Probabilistic Entropy Concept

**DOI:** 10.3390/ma15103587

**Published:** 2022-05-18

**Authors:** Rafał Bredow, Marcin Kamiński

**Affiliations:** Department of Structural Mechanics, Lodz University of Technology, 90-924 Lodz, Poland; rafal.bredow@dokt.p.lodz.pl

**Keywords:** relative entropy, stochastic finite element method, stochastic forced vibrations, generalized stochastic perturbation technique, Monte Carlo simulation, first order reliability method, Hilber-Hughes-Taylor method

## Abstract

This work aimed to analyze the reliability of a steel hall that was recently erected in central Poland subjected to dynamic wind excitation using the stochastic finite element method. Reliability analysis was completed using the relative entropy concept delivered by Bhattacharyya and contrasted with the first-order reliability method recommended by the engineering design codes. Bhattacharyya probabilistic relative entropy was additionally rescaled in this study to fit the demands and recommended admissibility intervals given in Eurocode 0. The finite element method study was carried out thanks to a discrete model created in the system ABAQUS 2019, while all further statistical and probabilistic computations were programmed and completed in the symbolic environment of MAPLE 2019. Contrary to most engineering analyses in steel structure areas, this study included the important warping effect while designing the hall ridges and the purlins. Dynamic structural responses were determined via the Hilber-Hughes-Taylor algorithm and their series were numerically obtained for a series of input uncertainty parameters representing several mechanical and environmental quantities. The generalized 10th order iterative stochastic perturbation technique was contrasted in this context with statistical estimators from the Monte Carlo simulations and numerical integration resulting from the semi-analytical approach. The key research finding of this study was an extremely good coincidence between the FORM indices and the rescaled relative probabilistic entropies for the given stochastic excitations, which additionally did not depend on a choice of one of the three proposed numerical approaches.

## 1. Introduction

Designing of modern and optimal steel halls as well as other slender high steel structures is still widely carried out with the use of the extreme quasi-static equivalent [1] of wind pressure. Such an equivalent is scaled with an enormously high and sometimes unjustified safety coefficient, but wind pressure has a dynamic and chaotic nature. This is due to many civil engineering code regulations, a lack of experimental data, as well as the widely accessible appropriate probabilistic numerical apparatus. Steel halls usually have such large external surfaces that uncertain wind pressure should be modelled not as a single random variable, but preferably as a random field [2]; however, it needs an a priori probability density definition or advanced experimental measurements leading to the determination of spatial and time correlation functions. Therefore, even if the stochastic finite element method according to any probabilistic method is applied [3], the wind action is adopted on the basis of time spectra presented in the literature and scaled with the use of some random variables of frequently Gaussian character. The extreme values or the generalized extreme value distributions are preferred in this role, but they are hardly applicable outside of the Monte Carlo simulation scheme, the relatively huge time and computer power cost of which usually excludes such a modeling.

The second issue of mainly practical importance is the warping effect in thin-walled metal structures, which may be even more important in aluminum alloy engineering applications. It is known from Vlasov theory of thin-walled beams [4,5] that some specific external loads may generate remarkable warping, leading to highly non-linear distributions of normal and reduced stresses throughout open cross-sections with slender webs. This is in a clear contradiction to the existing designing codes (see Eurocode 3 at least [6]), where an assumption of linear normal stress distribution in various profile cross-sections plays an important role. Further, the FEM-based designs of civil engineering structures using warping-enriched beam finite elements are even now extremely scarce. Their new implementations are still being proposed [7], while some stochastic analyses in this area seem to still be unique even now [8,9]. However, a huge number of possible uncertainties appearing in modern thin-walled metal or composite structures leads to a frequent and realistic necessity of reliability assessment, which demands some stochastic computer methods for both static and dynamic structural responses.

On the other hand, it is well known that neither probabilities nor probabilistic moments are the only method to analyze uncertainty and stochasticity in structural mechanics. This can be done as well by using the probabilistic entropy apparatus, which is an extension and generalization of the entropy available in thermodynamics. An original concept of probabilistic entropy was proposed first by Johann von Neumann and was formerly introduced and defined as some universal measure of uncertainty (or disorder) by Shannon in 1948 [10]. This quantity was and still is most frequently applied in information theory [11], cryptography, and computer science, but its sporadic usage in mechanics is connected with the maximum entropy principle (MEP) and Bayesian method [12]. Such an approach appeared to be quite efficient to study uncertainty propagation in elasto-dynamics [13] and even to solve some optimization problems in dynamics [14]. However, all these studies do not contain direct calculation of probabilistic entropy values except for simple illustrative educational examples. MEP is used to justify probability distribution choice following maximization of the entropy. One of the powerful numerical methods necessary for the probability tails (and their logarithms) is undoubtedly Monte Carlo simulation, which in crude or interval versions [15] may enable even extremely advanced physical system simulations. Determination of the entropy (or uncertainty) propagation may be helpful in stochastic structural dynamics [16], structural health monitoring [17], and also for a prediction of the earthquake hazards [18]. It is also used in uncertainty propagation studies for computer simulation and homogenization of composite materials [19,20].

Moreover, an idea of the relative entropy appeared quite shortly after the publication of the original Shannon theory. Probabilistic relative entropy has been developed in probability theory to study probabilistic divergence of two different (univariate or multivariate) distributions [21], where Kullback-Leibler, Bhattacharyya, Hellinger, Shannon-Jensen, Tsallis, and some other approaches have been proposed. Nevertheless, their applications in probabilistic engineering mechanics and particularly in stochastic reliability assessment is definitely scarce.

Therefore, the main problem solved in this study was the stochastic finite element method analysis, reliability assessment, and relative entropy propagation for the steel hall optimally designed, manufactured, and recently erected according to the demands of Eurocodes. This aim was achieved using three concurrent probabilistic methods, namely, the iterative generalized stochastic perturbation technique based on general order Taylor expansions, the Monte Carlo simulation method based on random generation and statistical estimators, and a semi-analytical methodology. The 10th order stochastic perturbation technique was applied in this study in conjunction with polynomial structural responses (up to the 10th order) recovered by using the weighted least squares method. Numerical accuracy of such an approach has been demonstrated before in [3], for instance. The last methodology has been implemented with the use of polynomial bases numerically recovered via the least squares method and further symbolic integration relevant to the basic probability theory definitions of the central moments. Reliability assessment was provided using two alternative approaches including the first-order reliability method (FORM), and thanks to the Bhattacharyya relative entropy concept, all these techniques were successfully programmed in the computer algebra environment of MAPLE 2019. The core finite element method study has been completed using cloud computing using the computer program ABAQUS [22,23], where 7 DOFs (degrees of freedom) beam elements capturing warping behavior has been engaged; Hilber-Hughes-Taylor algorithm has assured high quality of dynamic response determination. This complex computational apparatus has enabled a verification (*i*) of the importance of the warping effects in the reliability-oriented design of steel halls as well as (*ii*) of the applicability of relative probabilistic entropy in the Eurocode-based determination of both reliability and durability of the exemplary steel structure. A coincidence of three probabilistic methods applied in this study is accordingly discussed in addition to an input uncertainty level in geometrical and mechanical structural parameters.

Finally, it should be underlined that the basic novelty of this work is in application of the relative Bhattacharyya entropy to study uncertainty propagation and reliability assessment of the structure subjected to a dynamic excitation. Another original aspect of this study is that such a dynamic reliability coefficient was efficiently rescaled to the level of admissible FORM reliability indices contained in Eurocode 0 to enable its further usage in practical reliability and durability verification.

## 2. Materials and Methods

### 2.1. General Overview and Geometry

The subject of this numerical study was the steel hall having a longitudinal and repeatable structural scheme, which has been finished in central Poland. These mainframes have been designed using tapered S355 I-beam profiles described by the yield strength of 355 MPa and elasticity modulus of 210 GPa. A constant distance between the mainframes has been set as 7.60 m, whereas the principal girders have 5 degrees of inclination from the horizontal direction and have been formed into the pitched roof. The purlins have been modelled as I-beams IPE200 and the ridge purlins as C200 profiles. All the geometrical dimensions of these cross-sections are presented in Table 1 below.

### 2.2. Computational Implementation of Thin-Walled Beam Finite Elements

It is commonly known that commercial FEM programs consider six degrees of freedom at each node of structural finite elements of a spatial frame; the warping effects are completely postponed in this way. This can be dangerous especially for the girders having huge slenderness because neglecting these effects limits the bearing capacity of steel profiles to only the Saint-Venant torsion resistance [4,5].

Warping effects are included into the Euler-Bernoulli elastic beam structural behavior by introduction of the seventh DOF at each node. It is introduced for the open and thin-walled cross-sections as the first derivative of the angle of a twist.

(1) $$\theta_{x}^{\prime }=\frac{d\theta_{x}}{dx}.$$

It denotes the rate of change of the angle of twist which can also be interpreted as the torsional curvature of the cross-section. When the expression (1) is included into the FEM formulation of the 3D finite element, its nodal displacements can be rewritten in local coordinates as [24,25,26]

(2) $$d^{e}=\left[u_{\tilde{x}j}u_{\tilde{y}j}u_{\tilde{z}j}\theta_{xj}\theta_{\tilde{y}j}\theta_{\tilde{z}j}\theta_{xj}^{\prime }u_{\tilde{x}k}u_{\tilde{y}k}u_{\tilde{z}k}\theta_{xk}\theta_{\tilde{y}k}\theta_{\tilde{z}k}\theta_{xk}^{\prime }\right]$$

The vector of nodal loads can be expressed similarly as

(3) $$f^{e}=\left[N_{j}Q_{\tilde{y}j}Q_{\tilde{z}j}M_{tj}M_{\tilde{y}j}M_{\tilde{z}j}M_{wj}N_{k}Q_{\tilde{y}k}Q_{\tilde{z}k}M_{tk}M_{\tilde{y}k}M_{\tilde{z}k}M_{wk}\right]$$

In the last expression, figure *M_t_* represents the moment of a twist at both ends of the finite element, which can be decomposed as

(4) $$M_{t}=M_{t}^{P}+M_{t}^{S},$$

where *M_t_^P^* is the moment relevant to the pure twisting state, while *M_t_^S^* corresponds to the warping effects. These two moments can be defined by the following expressions [4,5]:

(5) $$M_{t}^{P}=G_{1}I_{t}\frac{d\theta_{x}}{dx},\;M_{t}^{S}=-E_{1}C_{M}\frac{d^{3}\theta_{x}}{dx^{3}}.$$

In addition to that, in expression (3), bi-moment *B_w_* is correlated with a torsional curvature of the given cross-section. It follows the formula

(6) $$B_{\omega }=-E_{1}C_{M}\frac{d^{2}\theta_{x}}{dx^{2}},$$

where the following expressions describing geometrical cross-section characteristics have been adopted:

(7) $$C_{M}=\overset{K}{\underset{j=1}{\Sigma }}\frac{E_{j}}{E_{1}}\int_{\Omega j}{\left(\phi_{M}^{P}\right)}_{j}^{2}d\Omega_{j}$$

(8) $$I_{t}=\overset{K}{\underset{j=1}{\Sigma }}\frac{G_{j}}{G_{1}}\int_{\Omega j}{\left(z^{2}+y^{2}+z{\left(\frac{\partial \phi_{M}^{P}}{\partial y}\right)}_{j}-y{\left(\frac{\partial \phi_{M}^{P}}{\partial z}\right)}_{j}\right)}_{j}^{2}d\Omega_{j}$$

Having computed the aforementioned internal forces in the given structure, one is able to recover normal and tangent stress distributions along the cross-section. This would benefit from a determination of the extreme reduced stresses and also a verification of the linear stress distribution in this section, validating the applicability of the designing formulas included in Eurocode 3 [6].

### 2.3. Finite Element Method Numerical Model

**Figure 1:**
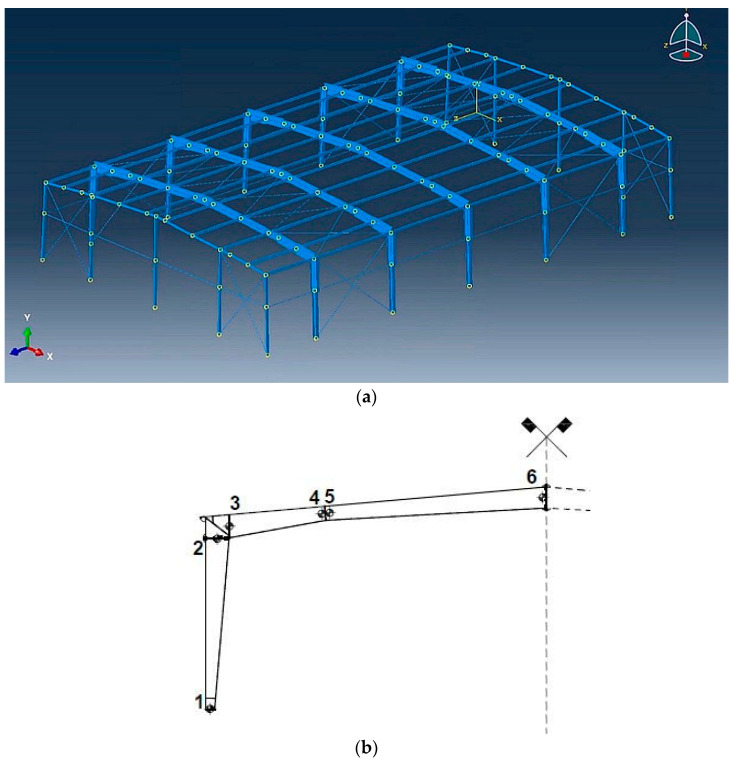
(**a**) Steel hall geometry overview. (**b**) Location of cross-sections described in Table 1.

The FEM discretization for this hall is fully 3D and has been completed in the system ABAQUS, see Figure 1; in most numerical studies, such a hall is modelled with the use of separate plane frames, for which stochastic analysis can be found in the literature [27]. As it is expected, a full 3D model benefits from the impact of the tensioned rebars and allows for a better optimization of steel cross-sections and their distribution. All connections between the columns and the ridges were set as perfectly stiff, and all the connections of these hall columns with the foundations were defined as the pin joints.

FEM analyses were performed using the beam finite elements B32OS consisting of 3 nodes, 7 degrees of freedom (DOFs) at each node, and quadratic shape functions. Warping deformation of all open section elements (columns and ridges) was included in all structural responses. Secondary elements were modelled as B32 elements which also have 3 nodes with quadratic formulation but have all 6 DOFs. Bracing elements were modelled as truss finite elements T3D2 which stands for 3D truss in space with quadratic formulation and 3 DOFs at each node. Overall, the FEM model contains 1457 finite elements and the total number of independent variables exceeded 16,900.

Computer implementation of B32OS finite elements brings additional and extremely attractive results for this contribution as the influence of the cross-sectional warp of finite elements on their stress-related ULS state is further inspected. It is commonly known that the twisting moment and warping moment coincide in the case of thin-walled open sections such as I-beams, and their mutual influence on the cross-sectional stress distribution might be significant. Most popular FEM commercial systems traditionally consider six degrees of freedom for beam elements in space and as a result, the warping effect is omitted. The aforementioned structures analyzed with the effect of only the Saint-Venant torsion resistance might be designed in an unsafe way due to the development of a cross-sectional warp. To include the warping behavior of members and further enhance the precision of the reliability estimation of the structure, the B32OS finite beam elements, implemented in the system ABAQUS, have been chosen. The governing equation of the position of each material point of cross-section in the B32OS finite element along its centerline at the given stage in deformation history is displayed in Equation (9).

(9) $$\overset{⌢}{x}\left(S,S^{\alpha }\right)=x(S)+f(S)S^{\alpha }n^{\alpha }(S)+\omega (S)\psi (S^{\alpha })t(S)$$

where the following notation was adopted:

x(S) —position of a point on the centerline of a finite element

$n_{\alpha }(S)$—unit orthogonal direction vector in the plane of the cross-section of beam, α = 1,2;

t(S)—unit vector orthogonal to $n_{1},\;n_{2}$;

$\psi (S^{\alpha })$—warping function such that ψ(0)=0 for the initial configuration;

ω(S)—warping amplitude; and

f(S)—scaling factor depending on the stretch of the beam.

### 2.4. Structural Load Cases

**Figure 2:**
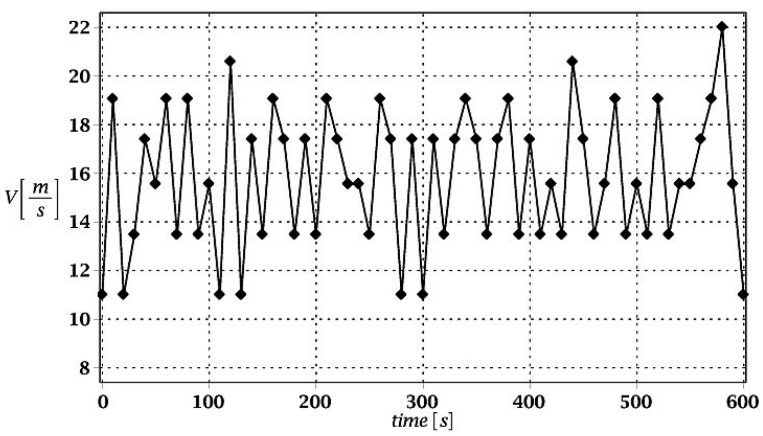
Dynamic wind spectrum.

Dead load of the roof insulation layers was introduced into the numerical model as the uniformly distributed line load acting on the purlins. Uniformly distributed snow load was applied to the structure [28] as a line load acting on all the purlins as well. Wind load action on the given structure was defined [1] as dynamic excitation within 10 min long time interval. The mean value of the wind speed (Figure 2) was introduced according to Eurocode involving terrain orography and location of the object, which finally resulted in dynamic wind pressure acting upon this structure.

According to Eurocode 1 and also fundamental research findings in wind engineering, its pressure is correlated with its velocity by the relation shown below

(10) $$q=\left[1+7\cdot I_{v}(z)\right]\cdot \frac{1}{2}\cdot \rho \cdot {\left(V\cdot c_{r}\cdot c_{o}\right)}^{2}$$

Dynamic responses of the investigated structure were further investigated in this study. State variables under consideration consisted of both SLS and ULS states described in Eurocode 0 and they include stress-related limit states of the structure as well as the global horizontal and vertical displacements.

### 2.5. Numerical Solution

A numerical solution was found thanks to simulations performed in the FEM system ABAQUS using a non-linear dynamic analysis, where the integration of equations of motion was carried out using the Hilber-Hughes-Taylor algorithm [29], and subsequent solutions were found with full Newton method. The stiffness matrix was self-updated after each increase in any iteration step of the FEM computations as geometrical nonlinearities in structural elements were accounted for. It would be interesting to extend the analysis presented with the limited deformations in steel structural element connections and also with some geometrical imperfections. The HHT algorithm is based on the discrete equation of motion (11) and describes nodal displacements and velocities at the given time step using the previous time step results presented below in Equation (12) as

(11) $$M{\ddot{x}}_{n+1}+(1+\alpha )C{\dot{x}}_{n+1}-\alpha C{\dot{x}}_{n}+(1+\alpha )Kx_{n+1}-\;\alpha Kx_{n}=F(t_{n}+\alpha \Delta t)$$

(12) $$\{\begin{array}{c} x_{i+1}=x_{i}+\Delta t\cdot {\dot{x}}_{i}+\left(1/2-\beta \right)\cdot {\left(\Delta t\right)}^{2}\cdot {\ddot{x}}_{i}\\ {\dot{x}}_{i+1}={\dot{x}}_{i}+\left(1-\gamma \right)\cdot \Delta t\cdot {\ddot{x}}_{i} \end{array}$$

Parameter *α* represents numerical damping and it can be taken within a range of [−0.3, 0.0]—the lower this parameter is, the higher the numerical damping. The lower bound of this parameter was employed in this study, providing maximum numerical damping. Further, the system ABAQUS automatically sets the time step in the method to achieve reliable numerical convergence. Nevertheless, a discrete series of structural responses was computed at each 5th second of the time domain; this returned 121 discrete data for each structural response. The governing equation of motion was solved for all discrete values of structural parameters separately listed in Table 2. These were (*i*) random wind velocity, (*ii*) random snow load, (*iii*) random elasticity moduli, and (*iv*) random thickness of webs and flanges of I-sections. Mean values of the chosen design parameters are underlined for a brevity of the presentation, and uncertainty in these parameters is introduced using Gaussian distribution.

### 2.6. Probabilistic Approach

Structural uncertainty numerical analysis of the given steel hall was delivered using the iterative generalized stochastic perturbation technique implemented in the system MAPLE [30,31] and using structural responses recovered by the FEM system ABAQUS. The 10th order perturbation scheme was implemented (as efficient enough due to the previous numerical experiments [3]). Polynomial approximations of structural responses were recovered by the weighted least squares method of up to the 10th order, so that at least 11 structural responses were needed at each time step. The input coefficient of variation was fixed as 0.10, which follows some experimental evidence [32]. Such polynomials have been proposed as the functions of structural elastic modulus and discrete time *t* in the following way:

(13) $$f(E;t)=\sum_{j=1}^{n}A_{j}^{\left(i\right)}\left(t\right)E^{j}\;\;,$$

It should be underlined that the most optimal order of this polynomial was detected via a statistical procedure, where both numerical error and variance of the FEM trial fitting were minimized, while the correlation factor of this fitting was maximized at the same time. Time-invariant analysis was undertaken at some arbitrarily chosen critical time step of random vibrations, and the input random dispersion was introduced as the additional parameter. In this way, the influence of input uncertainty on output statistical scattering is additionally discussed, where various orders of approximating polynomial bases were verified in this context.

These random structural responses were found using the iterative generalized stochastic perturbation technique (SPT) contrasted with Monte Carlo simulations (MCS) and semi-analytical method (SAM). This probabilistic approach aims to approximate random structural responses by their expectations and higher-order probabilistic characteristics so that the structural reliability can be estimated due to the Cornell idea. As it is known, the stochastic perturbation technique is based upon classical Taylor expansion series, which in case of a single input variable, may be rewritten as follows [3]:

(14) $$f(E)=f^{0}\left(E^{0}\right)+{\sum_{j=1}^{n}\frac{\varepsilon^{j}}{j!}\frac{\partial^{j}f}{\partial E^{j}}|}_{E=E^{0}}\cdot {\left(E-E^{0}\right)}^{j}\;\;\;,\;$$

where a superscript 0 is adjacent to the mean value of the given input random parameter and *ε* stands for the perturbation parameter (taken traditionally as equal to 1, also in this study), while *n* represents the order of the perturbation expansion. This expansion shown in Equation (6) is inserted into integral definitions of the central probabilistic moment of the *p*th order in form as shown below:

(15) $$\mu_{p}(R(E))=\int_{-\infty }^{+\infty }{\left(R(E)-E\left[R(E)\right]\right)}^{p}\;p_{E}(x)dx.$$

Probabilistic approaches proposed in this study use random polynomial bases shown in Equation (5). These polynomial bases were (*i*) used in conjunction with direct integration using classical probability integrals with Gaussian PDF kernel, (*ii*) implemented in the stochastic perturbation approach, and (*iii*) implemented in the random number generator and statistical estimators in Monte Carlo simulations. Additionally, the alternative reliability concept was proposed. Let us adopt the following expression for the reliability index estimation [33]:

(16) $$\beta^{\prime }=\int_{-\infty }^{+\infty }\sqrt{p_{R}\left(x\right)p_{E}\left(x\right)}\;dx,\;$$

where *p_R_(x)* and *p_E_(x)* define a probability function associated with structural resistance and a probability function related to structural effort, respectively. It can be derived analytically that this index may be expressed as [34]

(17) $$\beta^{\prime }=\frac{1}{4}\frac{{\left(\mu_{R}-\mu_{E}\right)}^{2}}{\sigma_{R}^{2}+\sigma_{E}^{2}}+\frac{1}{2}\ln\left(\frac{\sigma_{R}^{2}+\sigma_{E}^{2}}{2\sigma_{R}\sigma_{E}}\right).$$

This is true when an assumption about Gaussian distributions for the structural resistance and effort can be justified from the engineering point of view (this is the basis of Eurocode 0). The design parameters *µ_R_* and *µ_E_* traditionally denote the mean values of the structural resistance and effort, respectively, while *σ_R_* and *σ_E_* are their standard deviations. The numerical analysis performed in the next section also demonstrates a comparison of this index with the values returned with the Cornell theory [35].

## 3. Numerical Results and Discussion

The series of numerical simulations performed within this study allow us to present, and further, to discuss the parameters computed for the SLS and ULS cases at the ridge of the mainframe as well as at the corner connection of the roof girder and the column.

### 3.1. Serviceability Limit State Analysis

**Figure 3:**
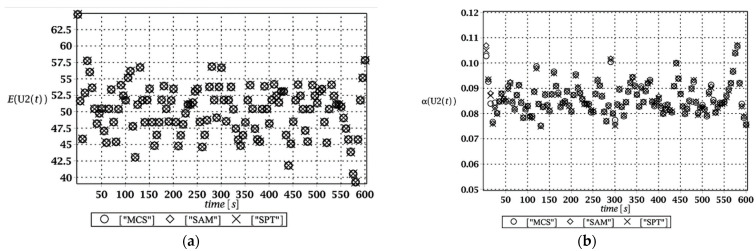
(**a**) The expectation of vertical displacement at the ridge of frame for random level of the snow; (**b**) its coefficient of variation.

**Figure 4:**
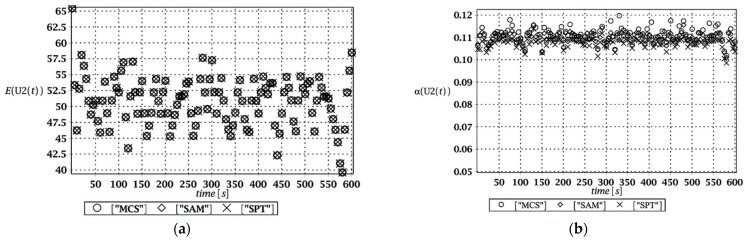
(**a**) The expectation of vertical displacement at the ridge of frame for random thickness; (**b**) its coefficient of variation.

**Figure 5:**
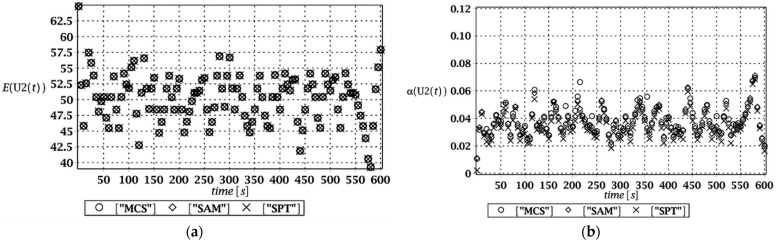
(**a**) The expectation of vertical displacement at the ridge of frame for stochastic wind pressure; (**b**) its coefficient of variation.

**Figure 6:**
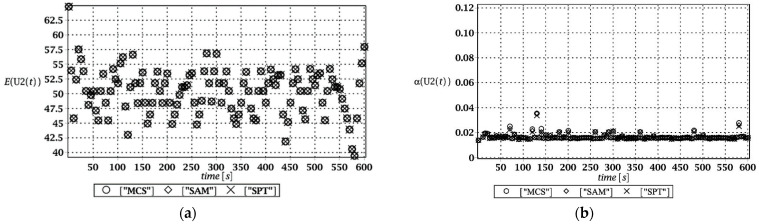
(**a**) The expectation of vertical displacement at the ridge of frame for uncertain Young’s modulus; (**b**) its coefficient of variation.

**Figure 7:**
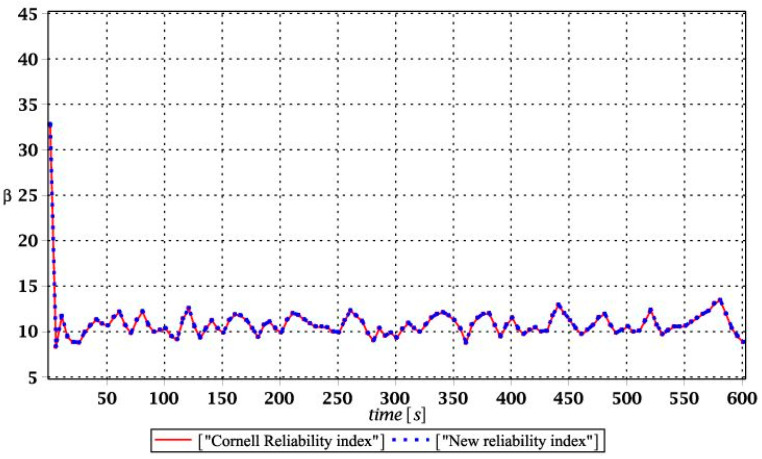
Comparison of dynamic reliability indices for uncertain snow load.

**Figure 8:**
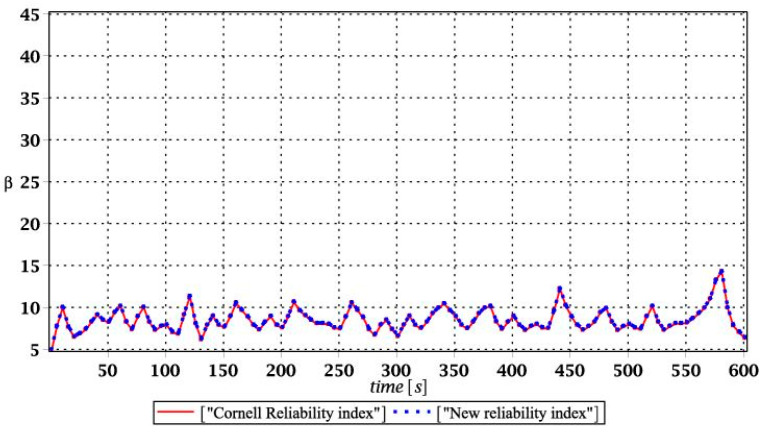
The comparison of dynamic reliability indices for uncertain thickness.

**Figure 9:**
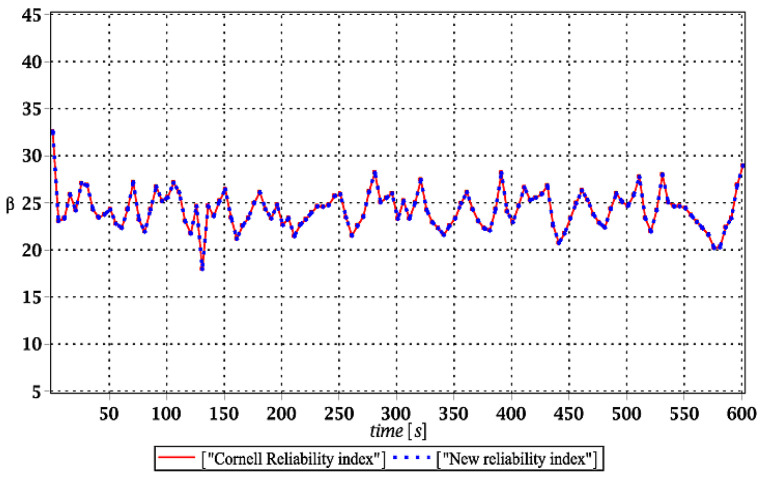
The comparison of dynamic reliability indices for uncertain wind pressure.

**Figure 10:**
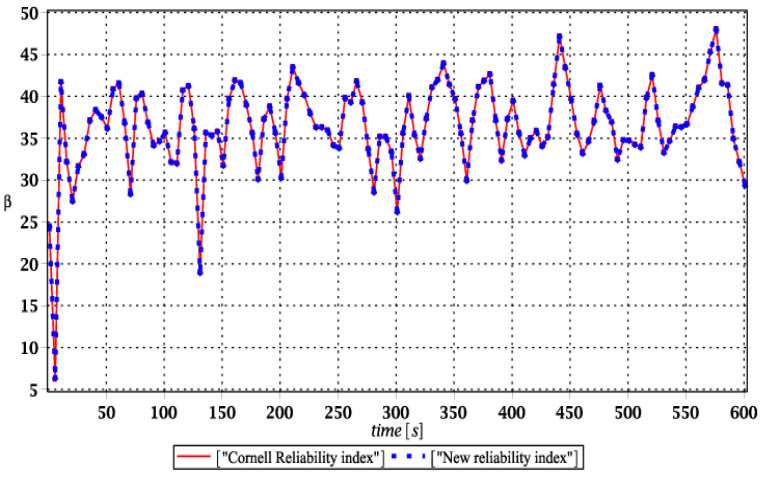
The comparison of dynamic reliability indices for uncertain Young’s modulus.

Probabilistic responses for the vertical displacement state at the ridge of the frame are shown in this subsection. They were calculated by the proposed methodologies and are presented in the graphs at each discrete time step of recovered structural responses. They include the expected values in Figure 3a, Figure 4a, Figure 5a and Figure 6a, accordingly, while the coefficients of variation are presented all in Figure 3b, Figure 4b, Figure 5b and Figure 6b. Both parameters are shown in the time domain and were computed using the three different probabilistic methods: Monte Carlo strategy (marked in the legends as MCS), semi-analytical method (abbreviated as SAM), and the stochastic perturbation technique (SPT). The next graphs contain a comparison of the FORM reliability index which follows Equation (16) [24] with the rescaled relative probabilistic entropy which follows Equation (17), cf. Figure 7, Figure 8, Figure 9 and Figure 10. The first two probabilistic moments and reliability indices were computed while randomizing: (*i*) snow loading, (*ii*) element thickness, (*iii*) wind pressure, and (*iv*) steel Young’s modulus.

These results clearly show that the most influential uncertainty source was the element thickness, which is important considering a possibility of its further reduction due to the corrosion processes. The second important was snow loading, which has been partially confirmed by the well-known failures of the steel hall systems. Less important were wind pressure randomization as well as the statistical dispersion of Young’s modulus. Quite expectedly, all three probabilistic methods engaged here perfectly coincided while comparing the first two probabilistic moments inherent in the SLS within the entire time domain.

Figure 7, Figure 8, Figure 9 and Figure 10 sequentially present the reliability indices, and they confirmed previous results that the most influential parametric uncertainty is seen in Young’s modulus and then in the ridge web thickness. This was concluded after the lower bound of this index was obtained within the given time interval; let us note that these lower bounds in both cases were reached at the beginning of the forced vibrations under consideration.

It is incredibly significant that both reliability methods—FORM and rescaled relative probabilistic entropy due to the Bhattacharyya model—returned exactly the same values and the same pattern in the given time domain. It seems that a rescaling procedure proposed for steel structures in static analysis [36] is valid and precise enough in dynamic response modeling. This means that relative probabilistic entropy may be used in reliability assessment, keeping demands and suggestions of the Eurocode 0. It is important because of the fact that FORM reliability index is valid for only Gaussian variables, while a general formula for this relative entropy (cf. Equation (16)) may be used to consider any uncertainty types. Perhaps other probability distributions would demand more sophisticated rescaling procedures and this would deserve a separate and more extended computer analysis. In addition to that, despite the Gaussian probability distribution of the input global uncertainty sources, all the resulting local structural responses had apparently non-Gaussian distributions.

### 3.2. Ultimate Limit State

**Figure 11:**
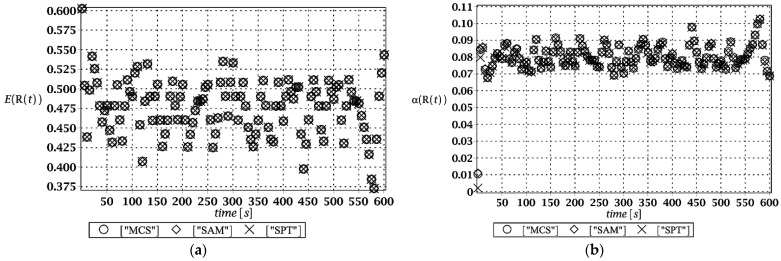
(**a**) The expectation of bearing capacity at the ridge of frame for uncertain snow load; (**b**) its CoV.

**Figure 12:**
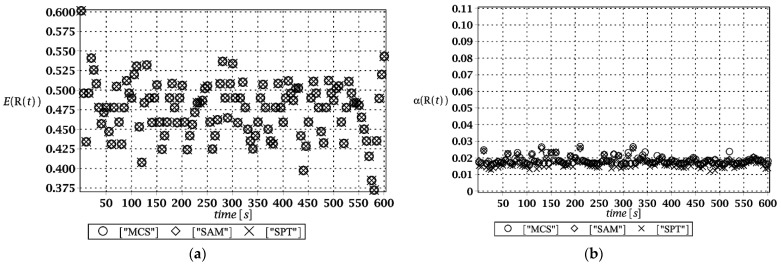
(**a**) The expectation of bearing capacity at the ridge of frame for uncertain thickness; (**b**) its CoV.

**Figure 13:**
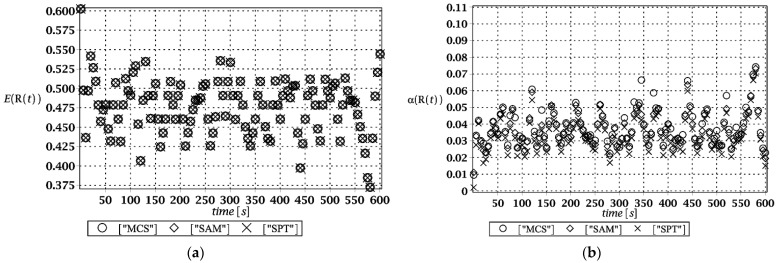
(**a**) The expectation of bearing capacity at the ridge of frame for uncertain wind velocity; (**b**) its CoV.

**Figure 14:**
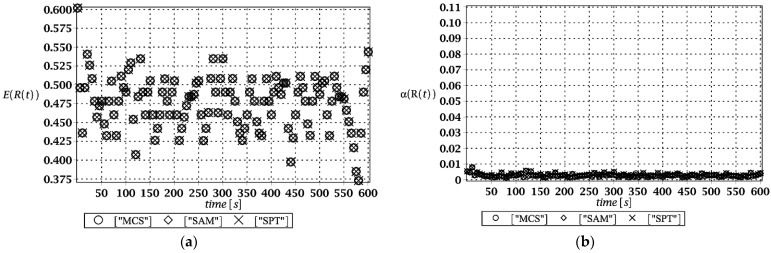
(**a**) The expectation of bearing capacity at the ridge of frame for uncertain Young modulus; (**b**) its CoV.

**Figure 15:**
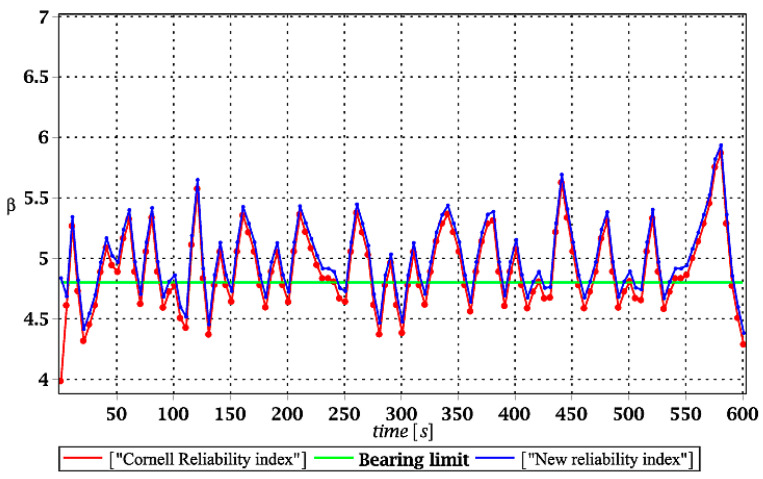
The comparison of dynamic reliability indices for uncertain snow load.

**Figure 16:**
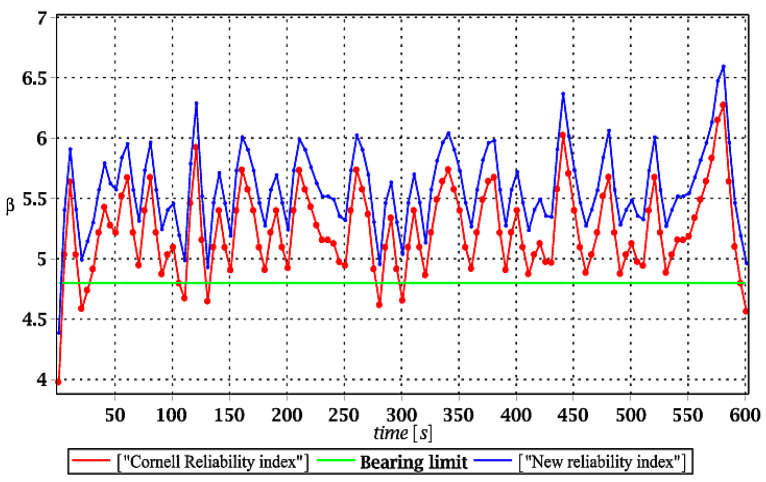
The comparison of dynamic reliability indices for uncertain thickness.

**Figure 17:**
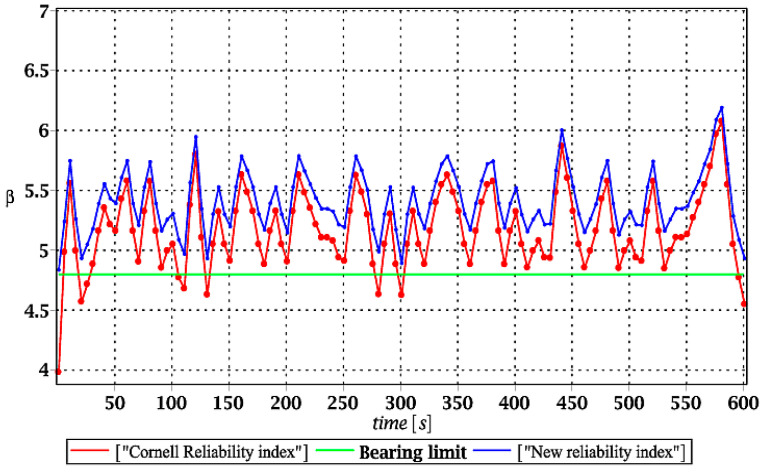
The comparison of dynamic reliability indices for uncertain wind pressure.

**Figure 18:**
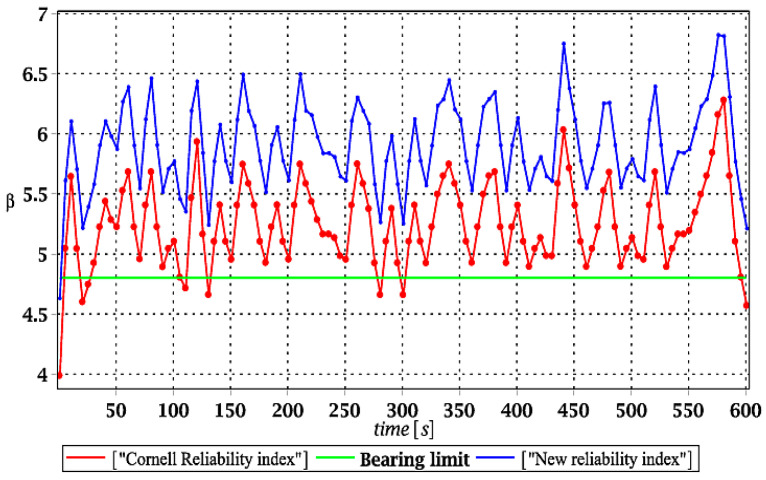
The comparison of dynamic reliability indices for uncertain Young’s modulus.

Similar to the previous subsection of numerical results, the expected values of the structural effort in the ridge for the ULS mode are presented in Figure 11a, Figure 12a, Figure 13a and Figure 14a. Most frequently, this effort is visualized in various computer CAD programs in percentages; however, decimal fractions were proposed here for a brevity of presentation. Additionally, their coefficients of variation (CoVs) are attached in Figure 11b, Figure 12b, Figure 13b and Figure 14b to check the impact of the input uncertainty. Finally, Figure 15, Figure 16, Figure 17 and Figure 18 report a contrast of the FORM and the relative probabilistic entropy in the reliability assessment for the ULS, including (*i*) snow cover thickness, (*ii*) ridge web thickness, (*iii*) mean wind velocity, and (*iv*) the ridge Young’s modulus. In addition, Figure 15, Figure 16, Figure 17 and Figure 18 contain the minimum level of structural reliability determined for the ULS marked as the “bearing limit”. Now, the evidence on reliability has been extended by marking the value corresponding to the deterministic upper bound on structural effort (marked with the green line in all these figures).

Generally, it was seen that our uncertainty analysis had some reverse results than for the SLS—the most influential statistical scattering was recognized in the case of the snow load (the highest resulting COVs), which was almost two times smaller for the wind load uncertainty. Remarkably smaller dispersions were noticed while randomizing web thickness and its Young’s modulus. Wind uncertainty exhibited high fluctuation within the given time domain, which was also observed in Figure 5b showing analogous COV while analyzing the SLS stochastic response.

Analysis of the results given further in Figure 15, Figure 16, Figure 17 and Figure 18 shows, first of all, that relative probabilistic entropy, even after the rescaling, returned systematically higher values than the FORM approach. These were the smallest in the case of the snow load (less than 1%), intermediate for wind pressure and ridge thickness, and largest in the case of Young’s modulus uncertainty (even close to 10%).

It seems that the FORM results were, in all these cases, more restrictive than the relative entropies; however, a comparison of these two series with deterministic full structural effort of the ridge showed that the Eurocode provision leads to unsafe dynamical safety. This is due to the fact that the FORM reliability index is smaller than the value corresponding to 100% structural effort, and this cannot be accepted in the optimal structural design. The rescaled probabilistic relative entropy avoided this unwanted effect in most cases, and the only exception was in Figure 15, where reliability indices for uncertain snow load are contrasted. This comparison means that relative probabilistic entropy application deserves further numerical investigations and that the rescaling procedure from entropy itself to an entropy-based reliability index should be more sophisticated. It may vary upon the character of the initial uncertainty and can have different multipliers for environmental actions, for the stochastic geometrical imperfections, and for material uncertainties. The conclusions could be drawn from further comparison of the relative probabilistic entropy presented here with the second-order reliability method (SORM), widely used in safety analyses [37].

## 4. Conclusions

(1) Numerical analysis delivered in this study confirmed satisfactory efficiency of dynamic response approximation using discrete polynomial bases. These bases appeared to be convenient for further probabilistic analyses, where three different computer techniques returned almost the same expected values, variances, and even higher order response statistics. Further, an application of the Bhattacharyya relative probabilistic entropy enriched with the additional scaling procedure was generally quite efficient in reliability analysis with the existing demands. This appeared to be true not only in static problems [34], but also in dynamics of structures with some uncertain parameters.

(2) Relative entropy exhibited similar behavior as the FORM reliability index, but particular numerical values were greater or equal to traditional engineering safety analysis. Generally, remarkably better coincidence was obtained while analyzing the serviceability limit state. A difference between relative entropy and the FORM reliability index for the ULS may depend on the given input uncertainty source—material or geometrical random parameters return higher discrepancies, while environmental actions are rather negligibly small. This difference could be further minimized by an additional reduction coefficient added to the rescaled relative entropy, i.e., by the ratio of both indices. The observed coincidence deserves further theoretical and numerical studies, especially in the context of material and geometrical structural nonlinearities [38,39] with some stochastic imperfections. Further comparison of Bhattacharyya relative entropy with other probabilistic divergency models such as Kullback-Leibler [40], for instance, may provide an interesting alternative to the existing FORM and SORM reliability algorithms.

(3) Warping functions, bi-moments, and torsional-bending moments resulting from the 3D ABAQUS model of the given steel hall together with their probabilistic characteristics may be efficiently used in stress analysis for the thin-walled cross-sections. Their knowledge would enable determination of their extreme values for different steel shapes and further reliability-based optimization of the profile choice for the specific static loadings or dynamic forcings in the skeletal thin-walled large-scale structures. The importance of SFEM modeling of such structures (having especially large-scale character and multiple geometrical scales) should be further investigated toward some hypothesis concerning limit slenderness, which disables traditional 6 D.O.F. beam elements and the demanding FEM model with the additional degree of freedom; special attention should be paid to the reliability of their joints and fasteners [41,42,43].

(4) Finally, it should be underlined that the results obtained in this study may find a direct application in structural health monitoring [44]. This problem appears important in engineering practice, taking into account a huge number of steel halls existing and still erected all around the world without any stochastic reliability or durability predictions [45,46]. It may also be valuable to apply the relative probabilistic entropy with the method based on the exact determination of probability distributions for the structural response [47], where the moments equations for the reliability index may not be necessary at all. Common application of the proposed approach with the so-called p-box methodology [48] may also be promising, because numerical values of the upper and lower bounds and the PDF formulas introduced in the p-box approach can increase the precision of interval simulation of both direct and relative probabilistic entropies.

## Figures and Tables

**Table 1:** Main girder cross-sectional properties.

Cross Section	1	2	3	4	5	6
Flange thickness	10 mm	10 mm	10 mm	10 mm	8 mm	8 mm
Web thickness	6 mm	6 mm	6 mm	6 mm	6 mm	6 mm
Web height	320 mm	840 mm	820 mm	500 mm	500 mm	750 mm
Flange width	270 mm	270 mm	230 mm	230 mm	200 mm	200 mm

**Table 2:** Discretization of the given uncertainty sources for the response function method.

No.	Elasticity Modulus	Peak Wind Velocity	Snow Load	Thickness of Webs and Flanges (Coefficient)
	GPa	m s^−1^	kN m^−2^	[-]
1	189.0	20.87	0.6480	0.900
2	193.2	21.10	0.6624	0.920
3	197.4	21.33	0.6768	0.940
4	201.6	21.56	0.6912	0.960
5	205.8	21.78	0.7056	0.980
6	210.0	22.00	0.7200	1.00
7	214.2	22.22	0.7344	1.02
8	218.4	22.44	0.7488	1.04
9	222.6	22.65	0.7632	1.06
10	226.8	22.86	0.7776	1.08
11	231.0	23.07	0.7920	1.10

## Data Availability

Not applicable.
